# Comparison of Retinal Thickness Measurements Using Optos Monaco and Heidelberg Spectralis OCT Across ETDRS Sectors in Normal Eyes

**DOI:** 10.3390/tomography11110124

**Published:** 2025-11-05

**Authors:** Kakarla V. Chalam, Lourdes Ceja, Rene Obispo, Minali Prasad, Anny M. S. Cheng

**Affiliations:** 1 Department of Ophthalmology, Loma Linda University, Loma Linda, CA 92354, USA; lourdes.ceja44@gmail.com (L.C.); rene_obispomd@yahoo.com (R.O.); 2 Chobanian & Avedisian School of Medicine, Boston University, Boston, MA 02118, USA; 3 Department of Ophthalmology, University of Florida, Gainesville, FL 32605, USA; annycheng@ufl.edu; 4 Department of Ophthalmology, Herbert Wertheim College of Medicine, Florida International University, Miami, FL 33199, USA

**Keywords:** Heidelberg Spectralis, optical coherence tomography, Optos Monaco, retinal thickness

## Abstract

**Simple Summary:**

Optical coherence tomography (OCT) is essential for quantitative assessment of retinal thickness in macular diseases. The Optos Monaco OCT, a new device, integrates wide-angle fundus photography with OCT for multimodal retinal imaging, unlike the established Heidelberg Spectralis OCT. This study assessed the Monaco’s OCT quality and its clinical utility as an alternative. We found that retinal thickness measurements from the Monaco and Heidelberg systems are not directly interchangeable due to systematic discrepancies. This highlights the critical need for consistency in the imaging device used for longitudinal patient follow-up to ensure reliable data. Clinicians should be cautious when comparing quantitative OCT data across different devices.

**Abstract:**

Purpose: To compare retinal thickness measurements obtained with the Optos Monaco and Heidelberg Spectralis optical coherence tomography (OCT) systems across 9 Early Treatment Diabetic Retinopathy Study (ETDRS) sectors in a cohort comprising normal eyes. Methods: Paired OCT scans from 64 eyes of 32 participants with normal retinal findings were acquired on both devices. Thickness measurements were obtained for the central subfield and the inner and outer sectors of the superior, nasal, inferior, and temporal quadrants. Outcomes included mean thickness, mean interdevice difference (Heidelberg minus Monaco), Pearson correlation coefficients, and Bland–Altman analyses. Scatterplots and Bland–Altman plots were constructed to evaluate agreement and assess potential interchangeability. Results: The Heidelberg Spectralis yielded significantly greater retinal thickness values than the Optos Monaco in all ETDRS sectors (*p* < 0.001), with mean differences ranging from +16.9 µm (outer superior) to +26.8 µm (inner superior). Pearson correlation coefficients indicated strong positive agreement (r ≥ 0.8) for the central subfield and most inner sectors, and moderate to strong positive agreement (r ≥ 0.5) in a single outer sector. Bland–Altman analyses demonstrated a statistically significant systematic bias favoring greater measurements with Heidelberg in most quadrants, with limits of agreement indicating clinically relevant variability. Although the relative agreement was high, absolute differences limit direct interchangeability. Conclusions: Optos Monaco and Heidelberg Spectralis exhibit strong linear correlation in retinal thickness measurements but show significant systematic differences. Interchangeable use requires the application of correction factors where segmentation variability may be greater.

## 1. Introduction

Optical coherence tomography (OCT) has become indispensable for the quantitative assessment of retinal thickness, serving as a cornerstone in the diagnosis, staging, and longitudinal monitoring of macular diseases such as diabetic retinopathy and age-related macular degeneration (AMD). Quantitative OCT metrics, particularly those based on standardized Early Treatment Diabetic Retinopathy Study (ETDRS) grids, facilitate the objective evaluation of disease progression and therapeutic response.

The Heidelberg Spectralis OCT is a commonly utilized device in retinal imaging, offering high-resolution visualization of retinal structures [1], however, certain technical and methodological factors may influence its diagnostic performance. In routine clinical practice, OCT acquisition is frequently accompanied by fundus photography, enabling structural–functional correlation in the assessment of retinal disease [2]. Recent advances in multimodal imaging have streamlined this workflow, allowing the simultaneous acquisition of OCT and fundus images to improve clinical efficiency and patient throughput.

The Optos Monaco represents one such integrated platform, combining ultra-widefield color imaging, spectral-domain OCT, and fundus autofluorescence in a single device. This capability offers a panoramic retinal view while concurrently obtaining high-resolution cross-sectional data. Nevertheless, inherent differences in segmentation algorithms between OCT systems may introduce systematic variation in retinal thickness measurements. Such discrepancies are of clinical and research relevance, particularly in multicenter trials, longitudinal monitoring, and situations where the interchangeable use of devices is considered.

Our previous work involving 34 healthy eyes demonstrated close agreement between the Optos Monaco and Heidelberg Spectralis OCT measurements [3]. However, that analysis was limited to manual measurements along the horizontal meridian at only 3 anatomical points, restricting its clinical generalizability. The present study expands on this work by systematically comparing retinal thickness measurements from the Optos Monaco and Heidelberg Spectralis OCT across all 9 ETDRS sectors in eyes without retinal pathology. The primary aim was to quantify interdevice agreement and assess the implications for interchangeability in both clinical practice and research applications where segmentation accuracy may be affected.

## 2. Materials and Methods

### 2.1. Study Design and Population

This retrospective observational study included healthy adult participants without known retinal disease who underwent imaging with both the Heidelberg Spectralis and Optos Monaco OCT systems. Institutional Review Board approval was obtained from the Loma Linda University Health Institutional Review Board (#5190290), and all procedures adhered to the tenets of the Declaration of Helsinki. Written informed consent was obtained as all imaging procedures constituted standard clinical testing.

Eligible participants were aged >18 years with normal retinal morphology on examination. Exclusion criteria included a history of intraocular laser or surgical intervention, intraocular pressure > 21 mm Hg, best-corrected visual acuity (BCVA) worse than 20/20, or refractive error exceeding ±0.50 diopters.

### 2.2. OCT Imaging and Data Acquisition

All OCT imaging was performed according to manufacturer-recommended protocols for each device. Technical specifications and acquisition details for both devices have been described previously [3].

The Heidelberg Spectralis system (spectral-domain OCT) employed automated retinal layer segmentation with an overlaid ETDRS grid for thickness analysis. The Optos Monaco, also a spectral-domain OCT platform, generated retinal thickness maps using its proprietary automated segmentation algorithm. Retinal thickness was defined as the distance between the internal limiting membrane and the retinal pigment epithelium, according to each device’s default processing parameters.

Monaco OCT images were generated with the Monaco OCT camera, which uses a blue target as the patient looks into the device. The operator of the OCT camera monitored the correct orientation without needing focusing given the built-in automatic scan-positioning. Similarly, the Heidelberg Spectralis OCT has a program to map the foveal umbo and the surrounding 1.5 mm^2^ area extending from the interface to the outer portion of the retinal pigmented epithelium-Bruch’s membrane complex. Automated segmentation was used to ensure that the exact same retinal region was analyzed by both Heidelberg Spectralis and Optos Monaco for an accurate comparison of retinal thickness. Automated segmentation systems are programmed to define basic features within retinal images. The segmentation algorithm applies a uniform set of rules to identify the fovea, optic disc, or other anatomical landmarks, allowing for a standardized definition of the corresponding measurement areas. By algorithmically detecting and delineating retinal layer boundaries, automated segmentation provides a consistent and objective method for defining the region of interest. Once the retinal boundaries are segmented, the software can then generate thickness maps based on these defined regions.

### 2.3. Retinal Thickness Extraction

Thickness values were extracted from all 9 ETDRS sectors: the central subfield (CST); 4 inner ring sectors—superior (SIM), nasal (NIM), inferior (IIM), and temporal (TIM); and 4 outer ring sectors—superior (SOM), nasal (NOM), inferior (IOM), and temporal (TOM). The CST represented the mean thickness within a 1-mm–diameter circle centered on the fovea. The inner and outer rings had radii of 1 mm and 6 mm, respectively, centered on the same location. Data from each device were exported to spreadsheet format and merged by unique eye identifier to enable direct paired analysis.

### 2.4. Statistical Analysis

Statistical analyses were performed using GraphPad InStat (version 3.06; GraphPad Software, San Diego, CA, USA) and Python (version 3.11; Python Software Foundation) with the pandas, matplotlib, and SciPy libraries. Continuous variables were expressed as mean ± standard deviation (SD). Paired *t* tests were used to compare the mean retinal thickness values between devices for each ETDRS sector. The mean interdevice difference (Heidelberg minus Monaco) was calculated in micrometers (µm). Pearson correlation coefficients (*r*) with corresponding 2-tailed *p* values were computed to assess linear association between devices. Agreement was further evaluated using Bland–Altman analysis, reporting the mean difference and 95% limits of agreement. Scatterplots were generated to visualize correlation, and Bland–Altman plots were constructed by plotting the interdevice thickness difference against the average thickness for each ETDRS sector to evaluate systematic bias and measurement variability.

## 3. Results

A total of 64 eyes from 32 participants with normal retinal morphology were included. The cohort demonstrated the minimal refractive error (mean ± SD, ±0.40 diopters).

### 3.1. ETDRS Subfield Analysis

The mean CST measured with the Heidelberg Spectralis OCT was 266.5 ± 17.5 µm, which was 22.3 ± 11.8 µm greater than the corresponding Optos Monaco measurement of 244.2 ± 19.5 µm (*p* < 0.001). In both devices, the greatest retinal thickness was observed in the superior inner macula (SIM) and nasal inner macula (NIM), with values of 339.8 and 338.2 µm, respectively, for Heidelberg Spectralis, and 312 and 313 µm, respectively, for Optos Monaco.

Across all ETDRS sectors, the Heidelberg Spectralis consistently yielded significantly higher thickness values than the Optos Monaco (*p* < 0.001). Mean interdevice differences ranged from +16.5 µm in the superior outer macula (SOM) to +27.8 µm in the SIM (Table 1).

### 3.2. Correlation and Agreement Between Optos Monaco & Heidelberg Spectralis OCT

**Figure 1:**
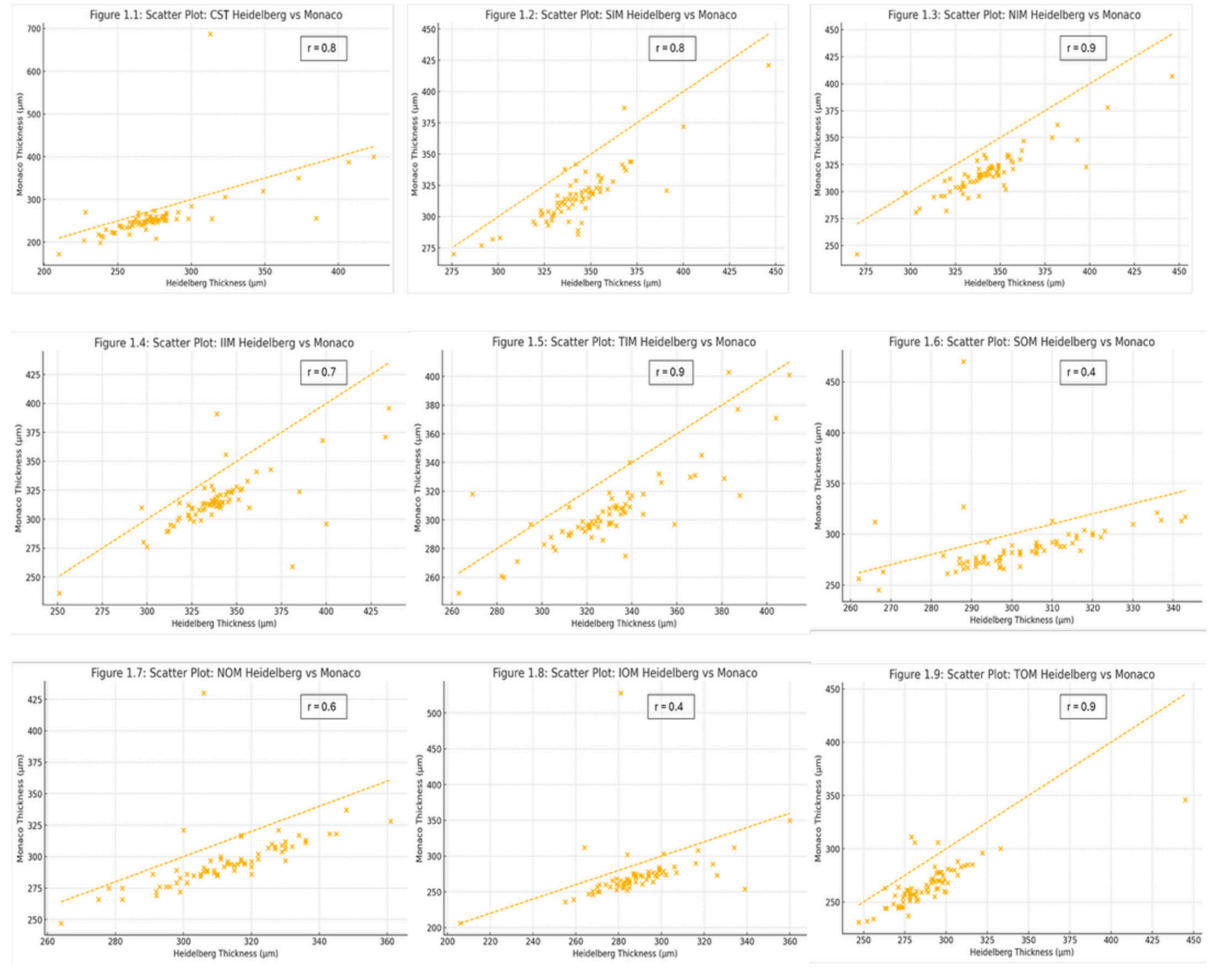
Scatter plots comparing the retinal thickness measurements obtained by Heidelberg Spectralis OCT (*x*-axis) and Optos Monaco OCT (*y*-axis) for each ETDRS sector (**1**–**9**). Each plot includes a dashed identity line representing perfect agreement (y = x) and correlation coefficient (*r*) from (*r* = Σ(*x_i_* − $\bar{x}$)(*y_i_* − $\bar{y}$)/√[Σ(*x_i_* − $\bar{x}$)^2^ Σ(*y_i_* − $\bar{y}$)^2^]). Points below the line indicate Heidelberg Spectralis measurements exceeding those from Optos Monaco.

Pearson correlation analysis demonstrated strong positive associations (*r* ≥ 0.8; *p* < 1 × 10^−11^) in the CST, SIM, NIM, temporal inner macula (TIM), and temporal outer macula (TOM) sectors, indicating consistent relative ranking of thickness measurements in these regions. Moderate positive correlations (0.6 ≤ *r* < 0.8) were observed in the inferior inner macula (IIM) and nasal outer macula (NOM). The SOM and inferior outer macula (IOM) exhibited weaker positive correlations (*r* < 0.5), likely reflecting increased segmentation variability in the perifoveal and more peripheral regions (Figure 1).

**Figure 2:**
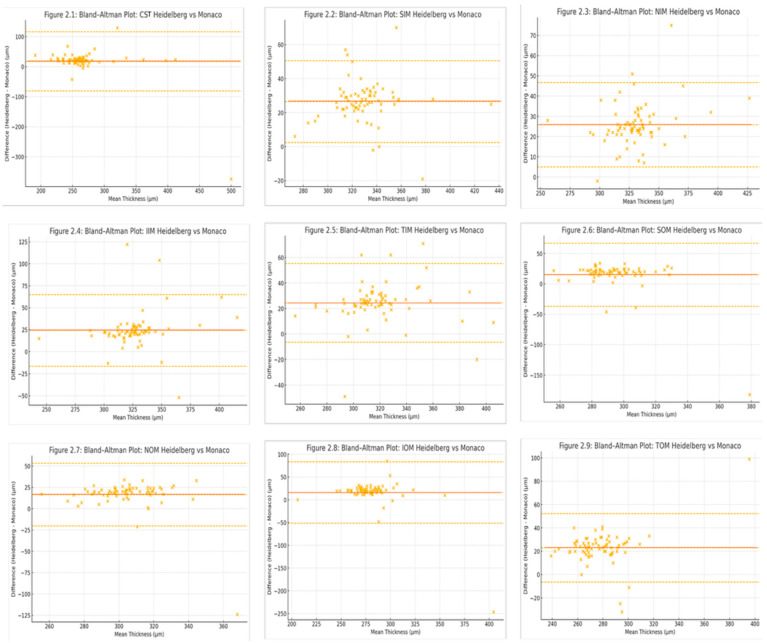
Bland–Altman plots for each ETDRS sector depicting the difference in retinal thickness between Heidelberg Spectralis and Optos Monaco (Heidelberg−Monaco) on the *y*-axis against the mean thickness of the two devices on the *x*-axis. The solid horizontal line indicates the mean bias; dashed lines represent the 95% limits of agreement (mean ± 1.96 × standard deviation) where the top and bottom dashed line are the upper and lower limits of agreement, respectively. Systematic positive biases were observed across all sectors. Wider limits of agreement in the central subfield (CST) and outer sectors reflect greater interdevice variability.

Bland–Altman analysis revealed systematic positive bias, with Heidelberg Spectralis measurements exceeding those of Optos Monaco by 16.5 to 27.8 µm on average (Figure 2). The 95% confidence intervals for the mean differences (bias) in all quadrants excluded zero, confirming statistically significant interdevice differences (Table 1). The limits of agreement were broadest in the SOM (−34.3 to +67.4 µm) and IOM (−52.0 to +87.7 µm), reflecting greater variability in the outer subfields. In contrast, narrower limits of agreement were observed in the CST and inner ring sectors, indicating more consistent interdevice performance in central macular regions.

## 4. Discussion

Optical coherence tomography (OCT), fundus photography, and fundus autofluorescence are among the most widely utilized imaging modalities in contemporary ophthalmic practice. In most clinical settings, these modalities are acquired using separate devices, a process that can prolong image acquisition times and reduce clinic efficiency [4]. In contrast, the Optos Monaco is capable of nonmydriatic acquisition of spectral-domain OCT, a 200° ultra–widefield color fundus photograph, and fundus autofluorescence in a single imaging session of approximately 90 s, potentially streamlining clinical workflow and enhancing patient throughput [5].

The primary aim of this study was to compare the retinal thickness measurements obtained from the Optos Monaco OCT system with those from the Heidelberg Spectralis, a device widely regarded as the reference standard for quantitative retinal imaging. Automated retinal segmentation was employed for both systems to minimize potential error introduced by manual caliper measurements. Our findings demonstrate that while strong correlations were observed between devices in the central and most inner ETDRS sectors, significant systematic biases and substantial variability limit the direct interchangeability of thickness values.

These results differ from our earlier investigation of 34 normal eyes, in which manual caliper-based measurements using the retinal map analysis protocol revealed no significant differences in most ETDRS subfields except for a higher CST with Optos Monaco compared with Heidelberg Spectralis [3] The present study’s findings also diverge from those of a British prospective study involving 268 patients with various retinal diseases, which reported no significant difference in image quality between Optos OCT platforms, including Monaco, and the Zeiss Clarus [6]. Such discrepancies are likely attributable to differences in measurement methodology. Automated segmentation, which detects retinal boundaries algorithmically, offers greater reproducibility and eliminates certain sources of observer bias inherent to manual caliper methods. However, it also introduces device-specific algorithmic variability.

Although the correlation between devices in many subfields was strong, Bland–Altman analyses revealed consistent positive bias, with Heidelberg Spectralis measuring greater retinal thickness in all sectors. The relatively wide limits of agreement, especially in the SOM and IOM sectors, suggest that outer macular regions present greater segmentation challenges and that thickness values in these areas should be interpreted with caution when comparing between devices. While such differences are unlikely to alter initial diagnostic impressions, they could influence longitudinal assessment, particularly in diseases where small changes in thickness are clinically meaningful.

**Figure 3:**
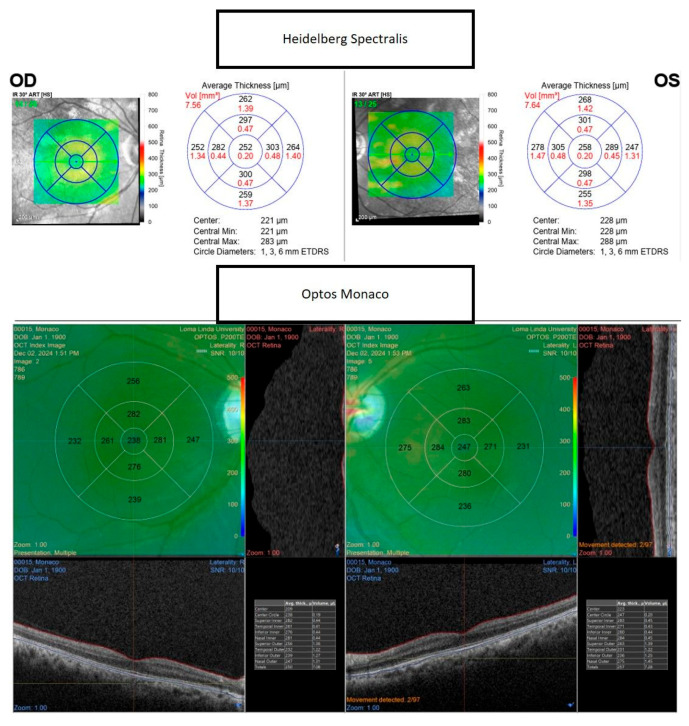
Representative images demonstrate greater retinal thickness values obtained with the Heidelberg Spectralis (**upper**) compared to the Monaco (**lower**) Optical Coherence Tomography (OCT) scan of the macula in healthy eyes.

The consistently greater retinal thickness values obtained with the Heidelberg Spectralis (Figure 3) are most plausibly attributable to differences in device-specific segmentation algorithms, scanning protocols, and hardware characteristics. The Heidelberg Spectralis offers an A-scan rate of 85,000 scans per second compared with 70,000 for Optos Monaco, a difference that may improve image stability and reduce motion artifacts [5,7]. The axial resolution of Optos Monaco exceeds that of Heidelberg Spectralis, potentially allowing finer boundary delineation, while its scan depth (2.5 mm vs. 1.9 mm for Spectralis) may influence segmentation by capturing deeper retinal structures [5,7]. These hardware and algorithmic differences underscore the importance of maintaining device consistency in longitudinal studies of macular disease, where subtle variations may alter treatment decisions. Automated segmentation, which delineates retinal boundaries algorithmically, provides greater reproducibility and mitigates certain sources of observer bias inherent in manual caliper measurements. Nonetheless, variations in imaging technology and acquisition protocols between devices—including differences in image quality, resolution, and scanning patterns—may influence the accuracy of automated segmentation. To promote consistency across platforms, automated segmentation algorithms are increasingly validated against multiple spectral-domain OCT devices to confirm their robustness in generating clinically meaningful metrics [8]. Refractive correction and scan quality during image acquisition are also critical, as positive defocus or reduced scan quality can significantly alter retinal nerve fiber layer thickness measurements. Accordingly, this study restricted inclusion to subjects with a refractive error within ±0.50 diopters and best-corrected visual acuity of 20/20 or better to minimize these potential confounders. Future investigations aimed at developing standardized acquisition and analysis protocols are warranted to reduce such biases and enable more reliable comparisons of retinal thickness across devices and studies.

Our findings align with those of a previous study comparing the Optos Monaco with the Zeiss VISUCAM PRO, which reported good agreement in vertical cup-to-disc ratio measurements in healthy and glaucomatous eyes [9], suggesting that agreement may depend not only on the imaging devices but also on the anatomical region and pathology under investigation. The weaker correlations and broader agreement limits observed in our study’s outer sectors support the hypothesis that structural irregularities and reduced signal quality in peripheral retina exacerbate segmentation discrepancies.

Although this analysis may be confounded by demographic and biometric factors such as age and sex, its strength lies in its direct comparison with Heidelberg Spectralis, a recognized gold standard in retinal imaging [1]. While both devices demonstrated strong relative agreement according to Pearson correlation analysis, there were significant absolute differences in the retinal thickness values they reported, particularly in peripheral macular subfields. Clinicians should avoid substituting measurements between these platforms without correction or calibration. Device-specific adjustment factors, or exclusive use of a single device for longitudinal follow-up, are recommended to ensure accuracy.

In this study, retinal thickness was measured under standardized conditions, using the same operator, identical equipment, and consistent environmental settings over a short interval. The cohort comprised 64 healthy eyes from 32 patients in a paired design comparing 2 devices, thereby minimizing intersubject variability. A power calculation for this paired design indicated that with 32 subjects, the study had 80% power to detect a difference at a 5% significance level, supporting the statistical validity of the results. Although repeatability testing was not performed in the present analysis, Bland–Altman methodology is widely used to assess agreement between measurements and may also inform repeatability when repeated measures are available. A larger prospective cohort incorporating repeatability testing will be necessary to draw more definitive conclusions. In addition, future research should incorporate longitudinal designs to determine whether interdevice variability remains constant over time or changes with disease progression. Additionally, multicenter studies involving a range of retinal pathologies could further clarify the conditions under which cross-platform measurements may be valid.

## 5. Conclusions

**Figure 4:**
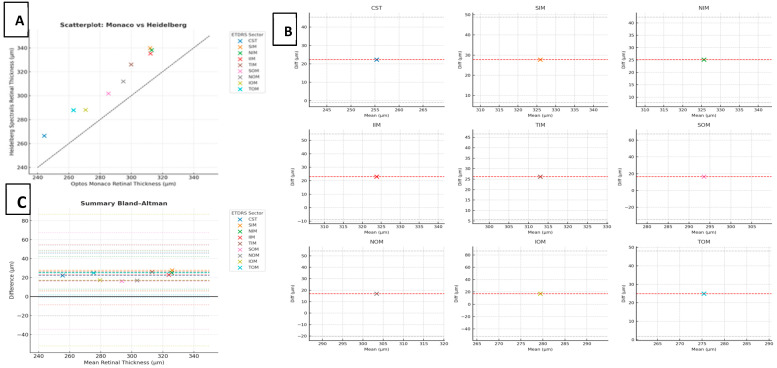
Summary of (**A**) scatterplot of retinal thickness measured by the Optos Monaco vs. Heidelberg Spectralis OCT across ETDRS sectors (n = 64 eyes). Each color represents a distinct ETDRS subfield. The dashed line indicates the line of identity (y = x). Strong correlations (*r* ≥ 0.8) were observed in CST, SIM, NIM, TIM, and TOM; moderate correlations (0.6 ≤ *r* < 0.8) in IIM and NOM; and weaker correlations (*r* < 0.5) in SOM and IOM. Summary of (**B**) Bland–Altman plots of retinal thickness measurements by ETDRS sector comparing the Optos Monaco and Heidelberg Spectralis OCT. Each panel displays the mean difference (red dashed line) and 95% limits of agreement (gray dotted lines) for a single ETDRS subfield. Positive bias indicates thicker measurements with Heidelberg Spectralis. Wider limits of agreement were observed in SOM and IOM, reflecting increased measurement variability in peripheral macular regions. Summary of (**C**) Bland–Altman plot overlaying all ETDRS sectors. Each color-coded point represents the mean difference in retinal thickness for a given ETDRS subfield plotted against the average thickness across devices. Colored dashed lines indicate sector-specific biases; dotted lines show the 95% limits of agreement. Greater variability was observed in the outer subfields (SOM and IOM) compared with the central and inner sectors, suggesting reduced interdevice consistency in the peripheral macula.

In summary, although the Optos Monaco and Heidelberg Spectralis OCT devices demonstrate strong correlation in central and inner macular thickness measurements, systematic bias, hardware and algorithmic differences, and greater variability in outer macular subfields limit their direct interchangeability (Figure 4). This line of research is clinically valuable because it advances the precision and standardization of quantitative OCT assessment. Moreover, our findings reinforce that clinicians should maintain consistency in the imaging platform used for serial follow-up to ensure accurate longitudinal comparisons, as switching between devices may introduce systematic discrepancies that could confound clinical interpretation and patient management. Consistent device use or application of validated correction factors is essential for accurate longitudinal monitoring. Future research is warranted to investigate how small measurement discrepancies from different devices might affect clinical decisions in patients with retinal pathology.

## Figures and Tables

**Table 1:** Summary of the key results for all 64 eyes.

Sector	Retinal Thickness Measured by Monaco (µm, Mean ± SD)	Retinal Thickness Measured by Heidelberg (µm, Mean ± SD)	Retinal Thickness Difference Between 2 Devices (µm)	Pearson r	*p*-Value	Limits of Agreement (µm)	95% Confidence Interval of Bias (µm)
CST	244.2 ± 19.5	266.5 ± 17.5	22.3 ± 11.8	0.8	1.8 × 10^−15^	−0.9~+45.5	19.3~25.2
SIM	312.0 ± 15.7	339.8 ± 18.5	27.8 ± 10.8	0.8	3.1 × 10^−16^	+6.6~+48.9	25.1~30.5
NIM	313.0 ± 16.6	338.2 ± 17.9	25.2 ± 8.8	0.9	5.6 × 10^−21^	+7.9~+42.4	23.0~27.4
IIM	312.3 ± 19.7	335.3 ± 22.4	23.0 ± 16.2	0.7	4.3 × 10^−11^	−8.6~+54.7	19.0~27.1
TIM	299.9 ± 16.3	326.1 ± 19.9	26.2 ± 10.4	0.9	3.4 × 10^−19^	+5.8~+46.6	23.6~28.8
SOM	285.3 ± 27.5	301.8 ± 15.3	16.5 ± 25.9	0.4	2 × 10^−3^	−34.3~+67.4	10.1~23
NOM	294.9 ± 23.2	311.9 ± 17.2	17.0 ± 19.0	0.6	2.4 × 10^−8^	−20.2~+54.3	12.3~21.8
IOM	270.7 ± 37.1	288.1 ± 20.0	17.3 ± 35.4	0.4	4.2 × 10^−3^	−52~+86.7	8.5~26.2
TOM	262.9 ± 17.2	287.9 ± 25.1	25.0 ± 11.8	0.9	1.7 × 10^−25^	+1.9~+48.1	22.1~27.9

Abbreviations: CST, central subfield thickness; IIM, inferior inner macula; IOM, inferior outer macula; NIM, nasal inner macula; NOM, nasal outer macula; SD, standard deviation; SIM, superior inner macula; SOM, superior outer macula; TIM, temporal inner macula; TOM, temporal outer macula; µm: micrometer; %, percentage; ~, to.

## Data Availability

The data presented in this study are available upon request from the corresponding author. The data are not publicly available due to their containing information that could compromise the privacy of the research participants.

## Abbreviations

The following abbreviations are used in this manuscript:

AMD	Age-related macular degeneration
CST	Central subfield thickness
ETDRS	Early Treatment of Diabetic Retinopathy Study
IIM	Inferior inner macula
IOM	Inferior outer macula
NIM	Nasal inner macula
NOM	Nasal outer macula
OCT	Optical coherence tomography
SD	Standard deviation
SIM	Superior inner macula
SOM	Superior outer macula
TIM	Temporal inner macula
TOM	Temporal outer macula
